# Vomiting of Pregnancy—Treatment with Wine of Ipecac

**Published:** 1879-05-20

**Authors:** D. B. Nesbit

**Affiliations:** Georgia


### *VOMITING OF PREGNANCY—TREATMENT WITH WINE OF IPECAC.*

BY D. B. NESBIT, M. D., OF GEORGIA.

John C——, (colored) called at my office on the 24th of March, 1879, to consult me on his wife's condition. He stated that she was sick and had been vomiting day and night for several days; would vomit all nourishment taken; stated that she did not know whether she was pregnant or not, but she did not come sick on "last moon." Knowing that she suffered considerably with vomiting of pregnancy, as I had treated her on a former occasion, I gave him several powders of oxalate cerium containing two grains each, with directions to give one every two hours until relieved, telling him to let me hear from her if she did not improve.

The following day I received a message from him requesting me to come to see his wife, as she was no better. On visiting her I found she was pregnant with second child, first month of gestation. She was considerably emaciated, pulse small and thready, 115 per minute; tongue dry and furred, and great tenderness on pressing over epigastric region. She stated she had been vomiting night and day for a week and would throw up everything she would eat; bowels constipated.

Having used minute doses of ipecacuanha in vomiting of drunkards with success, I determined to try it in her case, and so ordered:

Wine of ipecacuanha........................ gtts. xvj.

Aqua fontis.................................. ℥ ij. M.

with direction to give one teaspoonful every hour, day and night, until vomiting ceased, telling her if she did not improve during the day to let me know.

Not having heard from her that day or the following, I concluded she was better and did not call again.

Several days after my visit I saw her husband, and he stated his wife was perfectly well, with exception of being quite weak; stated she was completely cured of vomiting before taking half of the medicine, and was free from pain about stomach. I have seen patient during the last few days. She stated she has had no return of the vomiting since my visit, and is now perfectly well. I have since used the preparation in nausea of pregnancy without vomiting, in which case it acted like magic.

While I do not claim originality in the use of ipecacuanha in vomiting of pregnancy, as Ringer recommends it, yet I trust some of my professional brethren will give it a thorough trial, and if they meet with the same success that I have, they will always give homeopathic doses of wine of ipecacuanha in this troublesome and sometimes obstinate affection.

